# Effects of Olfactory and Auditory Enrichment on the Behaviour of Shelter Dogs

**DOI:** 10.3390/ani10040581

**Published:** 2020-03-30

**Authors:** Veronica Amaya, Mandy B. A. Paterson, Clive J. C. Phillips

**Affiliations:** 1 Centre for Animal Welfare and Ethics, University of Queensland, White House Building (8134), Gatton Campus, Gatton, QLD 4343, Australia; c.phillips@uq.edu.au; 2 Royal Society for the Prevention of Cruelty to Animals, Queensland, Brisbane, QLD 4076, Australia; mpaterson@rspcaqld.org.au

**Keywords:** dog, behaviour, shelter, arousal, stress, lavender, dog appeasing pheromone (DAP), music

## Abstract

**Simple Summary:**

Shelters are stressful environments for dogs, and this can lead to increased arousal levels, resulting in behaviour and welfare issues. Reducing arousal levels may be achieved with targeted sensory environmental enrichment. We exposed dogs to three different stimuli to reduce arousal and compared responses to these with a Control group. Dogs exposed to any of the stimuli vocalised less than the Control, and when exposed to Music and DAP they showed more resting behaviours than the Control. These behaviours are associated with increased relaxation and less arousal, and therefore these stimuli appear appropriate for use in a shelter environment. The results suggest that small changes to the environment of shelter dogs can have positive effects on their behaviour, which could help improve the quality of their experience while in care.

**Abstract:**

Shelter environments are stressful for dogs, as they must cope with many stimuli over which they have little control. This can lead to behavioural changes, negatively affect their welfare and downgrade the human-animal bond, affecting re-homing success. Arousal is evident in their behaviour, particularly increased activity and frequent vocalisation. Environmental enrichment plays an important role in reducing arousal behaviour, either through direct physiological effects or by masking stressful stimuli. The present study focused on sensory environmental enrichment, using olfactory and auditory stimuli under shelter conditions. Sixty dogs were allocated to one of four treatments: three types of enrichment, Lavender, Dog appeasing pheromone (DAP) and Music, and a Control group. Stimuli were applied for 3 h/d on five consecutive days. Dogs exposed to DAP lay down more, and those exposed to Music lay down more with their head down, compared to the Control. Those in the Control stood more on their hind legs with their front legs on the exit door, compared to those exposed to Music and DAP, particularly if they had only been in the shelter for a short time. They also panted and vocalised much more than dogs in the three enrichment treatments, which tended to persist during the 4 h period post treatment, and in the case of vocalisation into the subsequent night. The study suggests that all three enrichments had some positive benefits for dogs in shelters, as well as being non-invasive and easy to apply in the shelter environment.

## 1. Introduction

Behaviour problems are a major reason for dogs to be relinquished to rescue shelters [1]. Even though shelter staff attempt to give these animals a good quality of life, shelters are inherently stressful environments [2,3,4]. Stress is defined as a state in which homeostasis is threatened by intrinsic or extrinsic negative effects known as stressors [5,6], examples of which are noise pollution, novelty, social stress, as well as an unpredictable and uncontrollable environment [2,4]. A complex range of physiological and behavioural adaptive responses restore homeostasis [5,6]. Behavioural responses consist of increased vigilance and arousal [5,6], which are key elements of the stress response [7]. Arousal is defined as a state of psychological and physiological activation resulting from the activation of the autonomic nervous and endocrine systems (amongst others). The level of arousal regulates the level of responsiveness to external stimuli [8].

Behaviourally, arousal results in increased sensory sensitivity and alertness; physiologically, it results in the production of corticosteroids and increased blood pressure and heart rate [8]. Intermediate levels of arousal are necessary to enhance performance, in contrast, too low or too high arousal levels will have detrimental effects and impair performance [8,9]. For example, hyper-arousal is considered a major concern for shelter dogs, as the time they spend in care can adversely affect their behaviour, and even dogs that enter the shelter without any behaviour issues have an increased risk of acquiring one over time [4]. This not only affects their welfare, it can also decrease their chance of being rehomed [3] and increase the possibility of euthanasia [4].

A possible useful tool to help reduce arousal levels is sensory environmental enrichment. This stimulates one or more of the dogs’ senses and should be easily applied in a shelter environment [10]. Auditory and olfactory senses are suitable targets for enrichment; with music, lavender and dog appeasing pheromone (DAP) having been the subject of research previously.

Music has psycho-physiological effects in humans and has been used previously as a form of therapy [11,12]. Some of the beneficial physiological responses include reduced blood pressure, heart rate and respiration rate [13], as well as being anti-anxiolytic [14,15,16]. Considering the positive effects that it has in humans, it is not surprising that classical music induces relaxation in chickens [17], Asian elephants [18], and Western lowland gorillas [19]. 

Several different types of auditory stimuli, including human conversation and various genres of music (classical, heavy metal, pop and especially designed/altered classical music) have been tested in shelter dogs. When exposed to classical music, dogs perform more behaviours associated with relaxation, e.g., resting, and fewer behaviours associated with arousal, e.g., vocalisation [20,21]. However, over the period of a week dogs can habituate to classical music if the same music is repetitively played [22]. In a study comparing different music genres (soft rock, motown, pop, reggae and classical music) changed daily, shelter dogs spent more time lying down in all of these except for reggae, when compared to a silent period [23]. Habituation was very limited. 

Another form of environmental enrichment is aromatherapy, which uses volatile essential oils that have physiological and psychological effects on the animal [24]. Lavender essential oil has been associated with improved psychological well-being and relaxation in humans [25,26,27,28,29,30,31,32], and with sedative effects and more relaxed behaviours in animals, including mice [33,34], pigs [35], dogs [36], horses [37] and sheep [38]. Shelter dogs exposed to lavender oil spent more time resting and less time walking around the kennel and barking, compared to other essential oils and a Control treatment with no olfactory stimulation [39]. 

Pheromones can also modify behaviour. Dog appeasing pheromone (DAP) is a synthetic compound based on the fatty acids secreted by the sebaceous glands in the mammary glands of bitches from 3–4 days after parturition to 2–5 days after weaning [40]. It has been tested in dogs with noise phobia [41], travel-related problems [42], house soiling tendencies [43] and anxiety-related behaviours in veterinary practices [44]. It worked best in puppies during training sessions, reducing fear and excitability, increasing sociability and making the puppies relaxed and better at learning during interactions with other puppies [45]. DAP has also had positive effects in shelter dogs when continuously applied through a diffuser for 7 days, with a reduction in the dogs’ barking amplitude and increased resting [46]. However, Frank et al. [47] argue that much of the scientific literature provides insufficient evidence of DAP’s effectiveness in reducing undesirable behaviours. 

Effects of enrichment on dogs may vary with their breed [48,49,50,51], age [20,48], sex [20,49,50,51,52], temperament [53] and prior experiences [49,51], since all of these factors influence their behaviour. For example, when challenged with stressful situations, male dogs show more tail wagging, higher posture but less paw lifting and changes of locomotion and posture than females [51]. When approached by a familiar person, female dogs show less ambivalent postures than males, and when undisturbed, they wag their tails more than male dogs [52]. Similarly, age may affect responses. Puppies have reduced cortisol responses and are more relaxed than juvenile/adult dogs when challenged [53]. Older dogs are less likely to explore their environment [48], sleep more during the day and less at night [54] and urinate more frequently [54]. However, some studies have not found differences in dog behaviour due to their sex and age [22,55,56]. It is also important to consider the lengths of dogs’ stay in shelters, since they may adapt and the environment may become familiar over time [57,58,59], with some [21,58,60] but not all [55,57] studies showing a reduction in activity over time. 

Although the stimuli discussed above have been demonstrated to have positive effects on the behaviour of shelter dogs, there has been no study that systematically compares different types of sensory stimuli in a shelter context. Therefore, the aim of this study was to compare the effects of music, lavender and DAP as enrichments on the behaviour of shelter dogs, and particularly to examine which reduces arousal the most. 

## 2. Materials and Methods 

### 2.1. Kennel Environment

**Figure 1:**
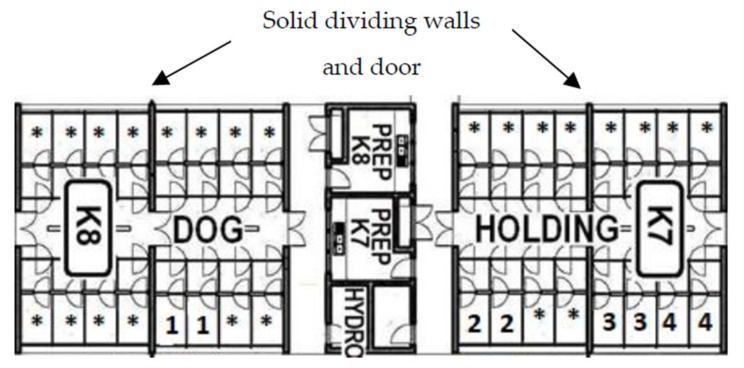
Kennels used for the measurement of the behaviour of dogs (*n* = 60) exposed to, Music, DAP, Lavender stimuli or a Control. K7 and K8 were the two blocks of dog kennels used for the study. 1: kennels for the Lavender treatment; 2: kennels for the Music treatment (separated from the other treatments by solid dividing walls); 3: kennels for the Control treatment; 4: kennels for the DAP treatment; *: kennels not holding a study dog.

This study was conducted at the Royal Society for the Prevention of Cruelty to Animals Queensland’s (RSPCA Qld) Animal Care Campus at Wacol, Brisbane, Australia, between August and November 2017. It occurred concurrently with regular shelter activities, such as routine cleaning, feeding and walking, and staff members and volunteers were always present in the kennel blocks. Each block consisted of 16 kennels (Figure 1), each divided into two rooms of 8 kennels (two rows of four) separated by a door. Dogs were individually housed in kennels, which had dimensions 1.6 × 4 m, and included a crate measuring 0.72 × 1.55 m and a bed. Both sides had plastic walls that prevented dogs from seeing each other. The back of the kennel had thin metallic bars from roof to floor, which permitted dogs to look outside and air to circulate. The front door had a solid section at the bottom and the same metallic bars from the top of the solid section to the top of the door. Each kennel had two water bowls that were refilled during the day when necessary. The dogs were taken for walks during the morning cleaning and in the afternoon while their kennels were spot cleaned. They were fed dry food twice a day. They were walked twice a day by volunteers for 10 min each time, and had occasional contact with volunteers at other times, except for the 3 h when they were exposed to the stimuli.

### 2.2. Subjects 

Research subjects consisted of 60 dogs, 35 males and 25 females, mean age 3.2 ± 2.4 years, range 6 months to 11 years, all desexed. The sources of these dogs were as follows: brought in by the shelter ambulance (*n* = 5), impounded by council (*n* = 26), surrendered by owners (*n* = 16), returned after a previous adoption (*n* = 6), strays (*n* = 4) and transferred from other shelters (*n* = 3). Dogs were categorised into 3 different age groups: young, ≥6 months and <2 years (*n* = 19), middle-aged, ≥2 years and < 4 years (*n* = 21) and old-aged, ≥4 years (*n* = 20). Their mean length of stay in the shelter was 45.9 ± 29.8 days, range 8 to 150 days. Most of the dogs in the study were cross bred and the primary breeds, which were identified by shelter staff on entry, are the following: Bull Arab (n = 12), Bull Terrier (*n* = 9), Kelpie (*n* = 5), English Staffordshire Bull Terrier (*n* = 4), Australian Cattle Dog (*n* = 3), Border Collie (*n* = 3), Boxer (*n* = 3), Bullmastiff (*n* = 3), Rhodesian Ridgeback (*n* =3), Shar Pei (*n* = 3), German Shepherd (*n* = 2), and one each of Bull Terrier, Hungarian Vizsla, Irish Wolfhound, English Mastiff, Great Dane, Louisiana Catahoula Leopard Dog, Pointer, Rottweiler, Saint Bernard and Siberian Husky.

On entry, all dogs had a veterinary clinical examination and a standardised behaviour assessment as described in Clay et al. [61]. Dogs with high arousal-related behaviours, such as air snapping, mouthing, attempts to bite lead or handler, excessive activity, constant vocalisation and over-reaction to other dogs, were selected for our study weekly by the RSPCA Qld Behaviour Team, based on their kennel behaviour and on information given by shelter staff working with the dogs. Dogs in need of immediate behaviour intervention as a result of hyperarousal, according to RSPCA Qld protocols, were not selected. RSPCA Qld staff were responsible for selecting the participant dogs and placing them in the study kennels; they were blind to the treatments and assigned dogs at random to each kennel as they became available. 

### 2.3. Study Design

Three forms of enrichment were applied in this study: Music (*n* = 14), Lavender (*n* = 15), Dog appeasing pheromone (DAP) (*n* = 16) and compared to a Control (*n* = 15). The kennels used for the Lavender treatment were in a separate kennel block to avoid other dogs in the study being influenced by the lavender, which potentially could spread to surrounding kennels. The other two enrichment treatments and the Control were set up in adjacent kennel areas divided by a wall and door, DAP and the Control in one half and Music in the other half, to prevent dogs in other treatments hearing the music. All kennels in the block were occupied throughout the study period. Dogs were exposed to the stimuli in their kennel for 3 h/d on 5 consecutive days. This commenced between 10.30 and 13.30 h, depending when their walk, breakfast and cleaning of the kennels finished. Dog behaviour was also observed in a post-treatment observation period from 14.00 to 17.00 h. At 16.30 h kennel duties ended. A final night period was observed between 18.00 and 09.00 h on the following day, except for day 5 when recoding finished at midnight. Our behavioural observations used cameras, detailed below, and were therefore completed without disturbing the dogs [62], providing a measure of the animal’s response to its surroundings, indicative of its stress response [22].

For the Music treatment, a solo instrument, the pianoforte (hereafter piano), was selected because it needs less neurological processing than multiple instruments [63], and a wide variety of tracks are available. The piano was the sole instrument, except in 6 tracks in which there was accompaniment by violins for part of the tracks. For the music selection, we used an automated filtering mechanism based on an audio analysis algorithm [64]. The programme employed a psychoacoustic approach to assess audio features of the tracks [65], generating a metadata file with the required track structure and music attributes, including rhythm, pitch and timbre. First a large number of songs (301) were downloaded from a common music platform (Spotify, www.spotify.com/, see Appendix A for list of tracks). In this overall set of tracks we applied filters - one readily quantifiable parameter (tempo, in beats per minute) and two qualitative parameters (valence and energy, both on scales of 0–1.0) describing the characteristics of the overall track’s sound. Only tracks with a tempo of 70 or fewer beats per minute were selected, in an attempt to entrain the dogs’ normal resting heart rate of 70–120 bpm [66] to a lower value. Only tracks with a valence from 0 to 0.5 were selected, thus the musical positiveness was less euphoric. Low valence is associated with low heart rate, respiratory frequency and electrodermal activity, all physiological markers of parasympathetic activity [16]. Tracks were selected with an energy rating of less than 0.2, reflecting low intensity and activity. Energetic tracks feel fast, loud and noisy, as is the case with Death Metal, compared with, for example, a Bach prelude [67]. While these parameters are based in human psychoacoustics, we extrapolated them to our canine population on the assumption that music has similar effects in dogs as in humans.

A selection of51 tracks that fit the above criteria were played for 183 min each day with random track selection order on a Motorola^®^ mobile phone connected to a Logitech^®^ speaker. Both were placed in a plastic holder hung on the crate’s door (in the middle of the kennel) to make sure the dog could hear the music. The music was played at 70 dBA, measured using a Digitech^®^ mini sound level meter at the beginning of the Treatment, as used previously [68,69]. Sound was also recorded in the kennels used for other treatments to ensure that the music was not audible, recognising that there is better auditory acuity in dogs, compared with humans, at high frequencies [70]. 

For the Lavender treatment, two ultrasonic diffusers (Select Botanicals, Gladesville, New South Wales, Australia) were placed in each kennel, one in the crate and one at the back of the kennel, to make sure the dog was exposed to the odour, regardless of its location in the kennel. Both diffusers were placed under milk crates to avoid being damaged or tipped over by the dog. The dilution was 4 drops of 100% organic Bulgarian lavender (*Lavandula Angustifolia*) (Select Botanicals, Gladesville, New South Wales, Australia) in 60 ml of water.

For the DAP treatment, 3 and 5 pumps of a synthetic analogue of the canine appeasing pheromone (15.72 mg/mL; Adaptil^®^, Ceva, Glenorie, New South Wales, Australia) were sprayed on a bandana worn by the dog, the dog’s bedding as recommended by the manufacturer and at three different point of the kennel’s floor (2 back corners and front door).

The Control did not receive any extra sensory stimulus.

### 2.4. Data Collection and Analysis

Mini cameras with charge-coupled devices and infra-red facility (Signet^®^, Electus Distribution Pty. Ltd., New South Wales, Australia) were fitted at the front and back of the kennels to record behaviour continuously (24 h/d during the 5 d of stimuli exposure). Behaviour was recorded and observed in three periods of the day: the Treatment period (3 h), 5 min observed every 15 min, i.e., 12 separate observations lasting 3600 s in total; the Post-treatment period (4 h), 5 min observations every 30 min, i.e., 8 separate observations lasting 2400 s in total; and the Night period, 5 min of each hour were observed, i.e., 16 separate observations lasting 4800 s in total. Behaviour coding software (Boris^®^ version 6.0.4 for Windows [71]) was used to record behaviours in an ethogram (Table 1), developed using previous literature and the researchers’ experiences of dogs’ behaviour in the shelter. Time values were then transformed into % values (duration of behaviour/total observation time × 100, in s). Some behavioural observations were missed because of technical issues with a camera, a dog was out of camera frame or absent when out for a walk (18 during the Treatment period, 0.5% of total observations, 283 in the Post-treatment, 11.8% of total observations,) and 783 in the Night, 16.3% of total observations). These were treated as missing values and taken into account when calculating the percentage of time that the behaviours were performed for.

### 2.5. Statistical Analysis

The behaviour data were statistically analysed using Minitab 18 software. A Principle Component Analysis was initially performed to inspect behaviours for similarity, which were combined if appropriate, as detailed in the Results section. 

Each of the three observation periods (Treatment, Post-treatment and Night) was analysed separately. Treatment effects on behaviour were analysed using a Mixed Effects Model, which was constructed using dog as random factor and dog number (entry time to the study), treatment and day as fixed factors. A more complex model including age and sex and their interactions with treatment and treatment x day was tested, but no significant interactions of these two factors with treatment were found, and therefore these factors were removed from the model. Length of stay was also tested but no significant treatment effects on behaviour were found, with the exception of standing at exit door, which is detailed in the results. Residuals were inspected for conformity to normality using the Anderson-Darling test. Square root transformations were used if necessary, to secure normal distribution of residuals. Differences between individual treatments were examined using Tukey’s test when treatment differences were detected. 

Even after creating combinations of behaviours, some still did not have enough data for statistical analysis. For these, the data from the five days were collapsed and treatment effects determined using a General Linear Model (GLM) constructed with dog number and treatment as fixed factors. The same normality-testing steps mentioned above were followed in the analysis of the data. The following behaviours were very rarely observed and were not analysed statistically: paw lift, lick nose/lip and play bounce.

## 3. Results

Using the PCA results, similar behaviours were combined as follows: pace repetitively + spin, pawing + scrabble at bars/door/wall, chew bedding + chew objects (bandana, monitors and milk crates/diffuser wires), roll on ground + circling, and tail medium + tail high. 

### 3.1. Treatment Effects on Behaviour During the 3 H Treatment Period

**Figure 2:**
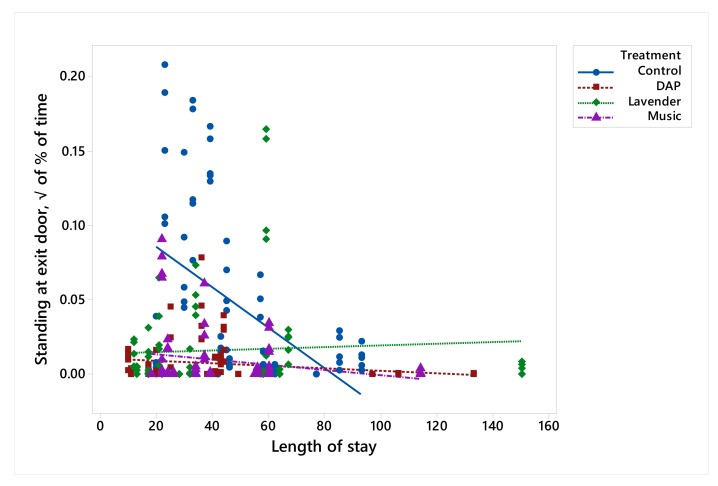
Relation between length of stay and √ % of time standing at exit door of dogs (*n* = 60) from the Lavender, Music, DAP and Control treatments during the 3-h treatment period.

Dogs exposed to DAP spent more time lying down, compared to the Control, with those in the Music and Lavender treatments intermediate (Table 2). Animals exposed to Music spent more time lying down with their head down, compared to the Control, with those in the DAP and Lavender treatments intermediate. There were no treatment effects on the time that dogs spent lying down with their head up. There was a trend for dogs in the Music and DAP treatments to stand and walk less than those in the Control (*p* = 0.08 and 0.09, respectively). Dogs in the Control spent more time standing on their hind legs with their front legs resting against the exit (‘standing exit door’ in the ethogram), compared to the dogs exposed to Music and DAP, with those exposed to Lavender intermediate. There was a significant length of stay x treatment effect for standing at the exit door (Figure 2). In the Control, dogs that had been in the shelter for only a short time spent longer performing this behaviour, whereas those in the enrichment treatments did not. 

Dogs in the Control spent approximately three to four times as much time vocalising as dogs in the three enrichment treatments. Dogs in the DAP treatment shook themselves the most, and they sniffed the ground more than the dogs exposed to Music, with those in the Lavender and Control intermediate. The dogs in the Control panted much more than dogs exposed to any of the enrichment treatments. They also excreted and moved their tail more, compared to Music and DAP, with those exposed to Lavender intermediate. There were no effects of treatment on position in the kennel.

### 3.2. Residual Treatment Effects on Behaviour during A 4 H Post-Treatment Period

Dogs that had been in the Control tended to spend less time lying down compared with dogs that had been in the three enrichment treatments (Table 3). They also tended to spend more time standing at the exit door (*p* = 0.08). They vocalised for longer, when compared to those that had been exposed to Lavender and DAP, with those exposed to Music intermediate. 

Dogs that had been exposed to DAP spent more time sniffing the ground when compared to those that had been exposed to Music, with Lavender and Control intermediate. Dogs that had been in the Control spent more time drinking water than those that had been in the Music treatment, with those that had been in the Lavender and DAP treatments intermediate. Those that had been in the Control also spent much more time panting than those exposed to Lavender, with those that had received Music and DAP intermediate. There were no effects of treatment on tail position/movement or dogs’ position in the kennel in the Post-treatment period.

### 3.3. Residual Treatment Effects on Behaviour during the Night Period

During the Night period, there was a trend for dogs that had been in the Control treatment to still vocalise more frequently than those that had been in the three enrichment treatments (*p* = 0.06) (Table 4). Dogs in the Lavender treatment groomed themselves more when compared to those that had been in the Music treatment, with those that had been in the DAP and Control treatments intermediate. 

## 4. Discussion

### 4.1. Treatment Period

The results of this study are in line with other studies where shelter dogs were exposed to similar stimuli: classical music [20,21,22], lavender [39] and DAP [46]. Dogs exposed to these stimuli displayed more restful behaviours and vocalised less in all the studies. The results from the present study suggest that sensory environmental enrichment (auditory and olfactory) can help to reduce arousal- in shelter dogs. 

During the Treatment period, when exposed to DAP dogs spent more time lying down compared to the Control, and when exposed to Music they spent more time lying down with their head down compared to the Control. These resting behaviours are associated with increased relaxation and lower arousal [73] and stress levels [20,21,39], and lying with their head down is particularly likely to be indicative of relaxation. Considering the busy shelter environment during the day, being able to rest more might be indicative of improved welfare [73].

Dogs in the Control treatment spent more time vocalising, a possible indicator of stress [74,75]. They also spent more time standing on hind legs with front legs resting against the exit (standing exit door) than dogs in the Music and DAP treatments, particularly if they had not been in the shelter for long. This behaviour may be escape motivated due to an interest in events happening outside of the kennel, but could also indicate boredom [76] or a quest for someone to come and see them. Dogs in this treatment also panted more, a behaviour that may suggest an elevated stress response in dogs [51,77,78]. It could also be related to the higher activity levels, or physiological responses to arousal increasing body temperature [51], with the dogs panting as a cooling mechanism [78]. Control dogs also excreted more than those exposed to Music; it has been reported that dogs kept under austere conditions and presumably experiencing higher stress levels show increased excretion [75,79]. 

Dogs in the DAP treatment shook themselves most, a behaviour that has been associated with acute stress [80] and the release of tension [75]. But considering that the other behaviours performed by dogs in this treatment are associated with more relaxing behaviours (more resting and less barking), the shaking could be due to some dogs finding the DAP bandana uncomfortable. Dogs from this treatment also sniffed the ground more than dogs exposed to Music; this is considered an investigatory behaviour [81] and they were possibly following traces of the pheromones as this was spread around the kennel’s floor.

The dogs’ location in kennel was not influenced by any of the stimuli, and nor was it affected by music in the work of Wells et al. [21], suggesting that they did not actively seek out the source of the auditory stimulation. 

Dogs in the Control treatment spent more time wagging their tails in comparison to dogs in the Music and DAP treatments. Tail wagging in dogs can be motivated by different things, such as play and appeasement, but other body signals can also infer the dog’s emotional state [82]. In this study, the increase appeared related to other behaviours connected to arousal, which is supported by other studies in dogs [9,72,75,83]. Beerda et al. [75] found that when dogs were challenged by situations such as poor housing, they increased tail wagging and had more changes in locomotion. 

Dogs exposed to Lavender showed the least behavioural differences when compared to the Control. This could be due to the lavender oil being very volatile and easily escaping the kennels, losing concentration and producing fewer anxiolytic effects in the dogs. It may be that concentrations near the floor were particularly low, diminishing any effects on the time that they lay down. Exposure to lavender oil, as a novel scent, can increase rather than decrease arousal in anxious animals, as documented by Hawken et al. [38] in a study in which nervous sheep exposed to lavender oil showed increased anxiety (more vocalisation, escape attempts and higher cortisol concentrations). In their study, calm sheep exposed to lavender showed behaviours related to reduced anxiety, such as less activity and vocalisation. Another possibility is that the lavender oil was not very effective producing behaviours associated with relaxation in the dogs tested.

The lower levels of tail movement (primarily wagging) and standing at the exit door in the Music and DAP treatments and the lower levels of vocalisation in the three enrichment treatments suggests that even small changes to their environment can help reduce arousal. Furthermore, the trend towards increased walking and standing in Control dogs suggests that they are less relaxed. 

### 4.2. Post-Treatment Period and the Night Period

During the Post-treatment period, dogs from the Control treatments drank more water than those exposed to Music. Increased water intake has been correlated with higher activity levels [51] which were performed by the Control dogs. 

**Figure 3:**
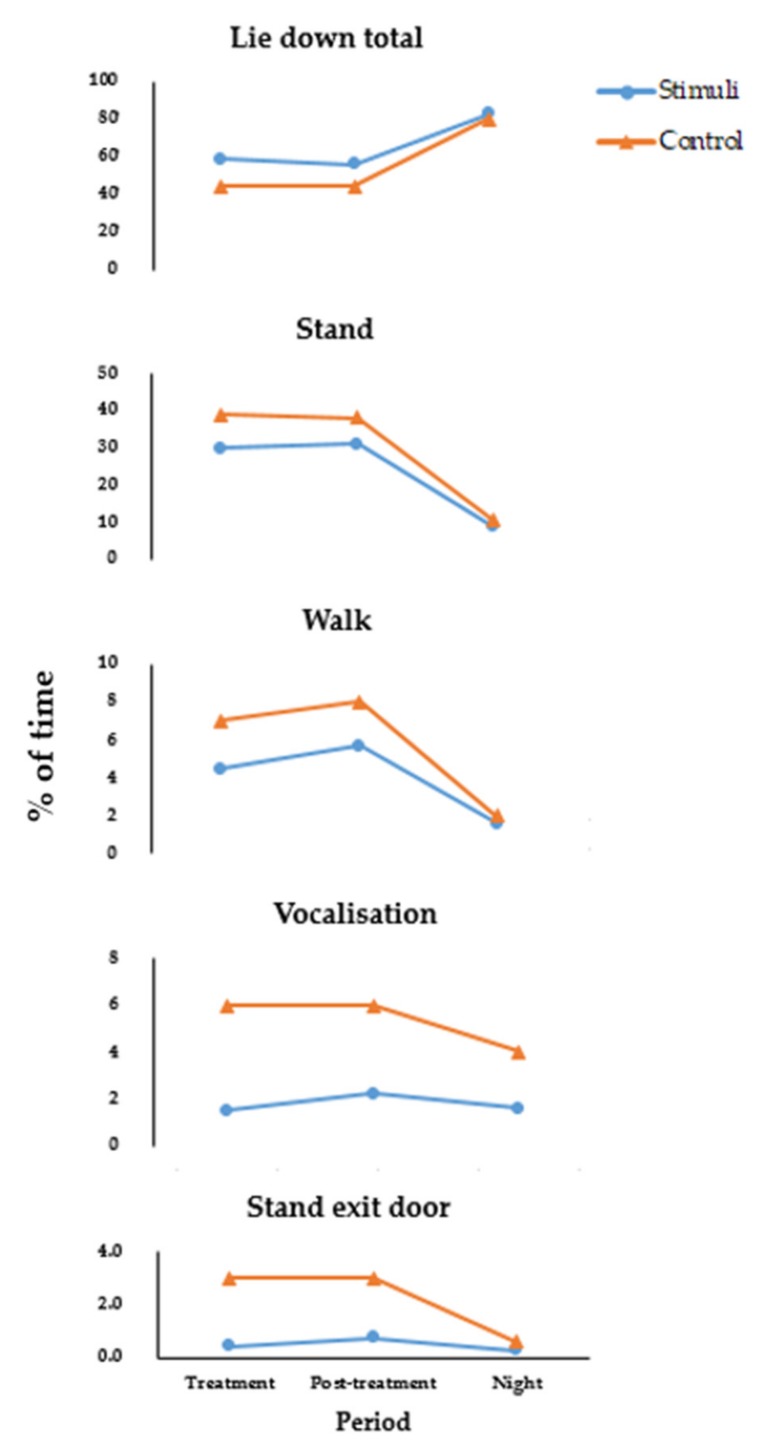
Proportion of time spent by dogs (*n* = 60) in the Control and amalgamated stimuli treatments (Music, Lavender and DAP), during the Treatment, Post-treatment and Night periods, demonstrating the elimination of these treatment effects except vocalization by the night period.

Dogs exposed to Lavender and DAP vocalised less than the Control treatments, indicating persistence of this behaviour, even into this Night period (Figure 3). High noise levels can affect the dogs’ auditory system and other physiological systems, such as the immune and endocrine systems [84]. They can also be detrimental for shelter workers’ auditory systems [84]. This major effect on vocalisation, a common stress response, could be utilised as a key stress indicator in shelters. Automatic detection may be possible, allowing attendants to monitor changes in stress levels in dogs in their charge. 

For the other behaviours that appeared to respond to stimuli, lie down total, stand, walk and standing at the exit door, the post-treatment effects were similar between the Treatment and Post-treatment periods, indicating some persistence of the beneficial effects of the enrichment, but these had mostly disappeared by the night time (Figure 3). 

During the Night period, dogs from the four treatments spent most of the time lying down and dogs in the Control barked less than during the other two observation periods. During this period the shelter has very little or no human presence, therefore there are not many external noises or stimuli to startle the dogs and arouse them. It seems that by this observation period most treatment effects had disappeared. 

### 4.3. Other Influences on Behaviour

The dogs’ responses to the stimuli remained similar during the five days of the trial, as there were no day x treatment interactions, suggesting a lack of habituation. Previous studies also failed to find habituation when dogs were exposed to lavender and classical music [21,39]. With music the extent of habituation may depend on the repetition frequency. As we had 51 tracks in our playlist, there was no repetition on the day, only across days and even then, the sequence was varied. Hence we can assume that there was insufficient repetition to produce habituation, as shown by Bowman et al. [22].

Even though there is evidence of age and sex affecting behaviour in dogs, we did not find that treatment effects on dog behaviour correlated to these variables, in line with other studies [22,55,56]. Length of stay evaluations have suggested that long shelter stays may produce changes in behaviour that could deter adopters, increasing the chances of further undesirable behaviours [85]. Contrastingly, Titulaer et al. [60] suggest that the dogs’ individual shelter experience may influence their behaviour, rather than the amount of time spent in care. Bowman et al. [22] found a relationship between time spent sitting and length of stay in a shelter, with dogs kenneled for more than three months spending more time sitting down when exposed to an auditory stimulus. However, as they did not find associations between length of stay and more relevant activities, such as lying down and standing, they concluded that behaviour responses were not influenced by length of stay. 

External variables must also be considered when trying to help shelter dogs to be more relaxed. Volunteers are extremely important for shelters, as the number of animals in care can be very large and to be able to give the animals the best care possible, many people are needed. This also means that there is a lot of human movement noise in the kennels, which can contribute to high arousal [86]. 

In this study, it was observed that when dogs were resting, noises like slamming doors would make them react immediately: walking around the kennel, jumping at the door, circling and barking. Dogs from all treatments reacted to this external stimulus, but these behaviours were exacerbated in the Control group. In Beerda et al.’s study [79], dogs in the most austere conditions reacted more actively to disturbances such as slamming doors. It is possible that some beneficial effects could be achieved in a shelter just by limiting the external stimulation that the dogs experience. For example, movement of people in and around kennels could be forbidden for several hours every day. The effect of such ‘quiet time’ would be worth investigating.

### 4.4. Limitations of the Study

We utilised only single levels of lavender release, a single volume for the music, and a single administration of DAP. It is quite possible that we did not use the optimal levels of any one of these treatments. Shelters are already noisy environments and if the extra acoustic stimulation is too loud, it could instead have a negative effect for the animals [87]. This could be equally true for lavender, as moderate doses are considered anxiolytic, but high doses cause sedation [88]. However, the lower incidence of vocalisation in all three treatments, along with behaviours associated with reduced arousal, e.g., lying down in the Music and DAP treatments, suggest that enrichment type may be more important than the level chosen. 

In future studies an initial period could be recorded, that could be used as a covariate to account for differences between dogs before treatment. However, our study took place in a working shelter, and having the dogs in the same kennel for 5 days was already challenging as it meant the dogs were unavailable for adoption during this time. The random distribution of dogs between treatments in our study could have produced biases in terms of age and gender distribution, however, as these were not related to treatment effects, it seems unlikely that this affected our results. 

## 5. Conclusions

Shelters can be very stressful environments and high arousal is a major issue that can stimulate undesirable behaviours in dogs in care. In our study, reductions in arousal-related behaviours were evident for dogs exposed to music and DAP for a 3 h period each day, and to a lesser extent dogs exposed to lavender for the same period. There was some persistence of responses over the 4 h post treatment, but in the subsequent Night period most responses had waned. There was no evidence of habituation to the enrichments over the 5 days of exposure for each dog. Shelters could consider using our music enrichment primarily, as is it the easiest and cheapest to apply. If music is used, the volume, type of music, extent of repetition and effects on care staff all need careful consideration. Even though DAP is expensive, it could still be justified for incoming animals showing high levels of stress, while they get used to their new environment. Lavender can only be recommended for more enclosed environments so that it persists, and dogs can get the full anxiolytic benefits of the essential oil. These types of stimuli are not invasive and easy to apply in a shelter environment, potentially having many positive effects in dogs experiencing high arousal. 

## Figures and Tables

**Table 1:** Ethogram used for the measurement of the behaviour of dogs (*n* = 60), with descriptors and references.

Behaviour	Description	Reference (If Available)
Pace repetitively	Dog repeatedly (>3 times) paces around kennel in a fixed route	[72]
Circling	Dog repeatedly (>3 times) walks around in small circles	[72]
Play bouncing	Dog repeatedly displays the play bow posture (>3 times)	[72]
Lie down-head up	Dog is reclining in a ventral position with its head up	
Sit	Hindquarters in contact with ground, front legs extended	[72]
Stand	Positioned with four feet in contact with ground and legs almost or fully extended	[72]
Walk	Forward movement with legs resulting in shift of whole body to a new position in enclosure	[46]
Lie down-head down	Dog is reclining in a ventral or lateral position, with a relaxed neck and head down	
Body shake	Dog shakes its whole body briefly as if drying itself	[72]
Paw lift	A forepaw is lifted off the ground and held there	[72]
Vocalisation	Sound emitted from the mouth, often repeated in quick succession	[46]
Yawn	Mouth opens wide for a period of a few seconds, then closes	[46]
Lick nose/lip	Tongue extends upwards to cover nose, before retracting into mouth	[46]
Pant	Mouth open with tongue extended accompanied with rapid breathing	[46]
Groom	Licking behaviours directed to own body	[72]
Body scratch	Use hind leg to scratch other part of the body	
Standing exit door	Standing on hind legs with front legs resting against the rear of the exit (at front of kennel)	[46]
Bars/ wall pawing	Using paws to reach through bars/against wall in a digging motion	[46]
Sniff ground	Walks with nose close to ground, presumed to be sniffing it	
Object play	Any vigorous or galloping gaited behaviour directed towards a toy or other object, including chewing, biting, shaking it from side to side, batting it with a paw	[72]
Drink	Imbibe water	
Excretion	Urination or defecation	
Tail medium/high	From −30° to +90° from horizontal	
Tail low	From −30° to −90° from horizontal	
Tail movement	Tail moving in any direction and speed	
Tail still	Tail is not moving	
Front of kennel	In front third of kennel	
Crate	At least 50% of the dog in crate	
Middle of kennel	In middle third of kennel	
Back of kennel	In back third of kennel	
Stand wall/bars	Standing on hind legs with front legs resting against wall/bars	
Door scrabble	Use front legs to scrabble at door while standing on hind legs	
Door pawing	Use front paw to hit door	
Wall scrabble	Use front legs to scrabble at wall while standing on hind legs	
Chew/play with bedding/bed	Chew or play with bedding or bed	[72]
Chew bandana	Chew neck bandana	
Chew monitor	Chew heart rate monitor attached to collar or monitor around chest	
Chew milk crate/wires	Chew milk crate or diffuser wires	
Roll on ground	Dog is upside down, rubbing its back against the ground	
Spin	Dog makes circular movement around itself	

**Table 2:** The behaviour of dogs (*n* = 60) exposed to Lavender, Music, DAP or a Control treatment, during the Treatment period.

Behaviour	Lavender	Music	DAP	Control	SED	F-Value	*p*-Value
Activity							
Lie down total, % of time	52.6 ^a,b^	61.3 ^a,b^	61.7 ^a^	44.4 ^b^	4.64	3.29	0.03
Lie down-head down % of time	38.7 ^a,b^	49.9 ^a^	43.6 ^a,b^	29.4 ^b^	4.72	4.46	0.008
Lie down-head up, √ % of time	3.52	3.13	4.01	3.58	0.337	1.24	0.31
% of time	12.4	9.79	16.1	12.8			
Stand, % of time	33.4	29.5	26.6	39.0	3.44	2.44	0.08
Walk, √ % of time	2.31	2.00	2.04	2.67	0.189	2.37	0.09
% of time	5.33	4.02	4.17	7.14			
Standing exit door, √ % of time	0.86 ^a,b^	0.55 ^b^	0.51 ^b^	1.67 ^a^	0.164	4.35	0.009
% of time	0.74	0.30	0.26	2.79			
Wall/door bounce, √ % of time ^1^	0.22	0.10	0.25	0.09	0.095	1.49	0.23
% of time ^1^	0.049	0.009	0.062	0.008			
Sit, √ % of time	1.90	1.16	1.65	1.39	0.316	0.81	0.49
% of time	3.60	1.35	2.74	1.93			
Vocalisation							
Vocalisation, √ % of time	1.12 ^b^	1.30 ^b^	1.27 ^b^	2.42 ^a^	0.291	6.90	0.001
% of time	1.26	1.70	1.61	5.87			
Other behaviours							
Body shake, √ events per hour	0.42 ^b^	0.30 ^b^	0.72 ^a^	0.33 ^b^	0.197	6.38	0.001
Events per hour	0.17	0.09	0.51	0.11			
Sniff ground, √ % of time	0.25 ^a,b^	0.09 ^b^	0.37 ^a^	0.27 ^a,b^	0.115	3.47	0.03
% of time	0.061	0.007	0.13	0.071			
Groom, √ % of time	0.52	0.37	0.67	0.42	0.199	1.65	0.19
% of time	0.27	0.14	0.45	0.18			
Drink, √ % of time	0.45	0.43	0.43	0.54	0.138	1.45	0.24
% of time	0.20	0.19	0.19	0.30			
Pant, √ % of time ^1^	0.48 ^b^	0.12 ^b^	0.36 ^b^	1.30 ^a^	0.267	7.26	0.001
% of time ^1^	0.23	0.01	0.13	1.69			
Yawn, √ events per hour ^1^	0.72	0.69	1.02	1.07	0.321	0.75	0.53
Events per hour ^1^	0.51	0.47	1.04	1.14			
Excretion, √ % of time ^1^	0.27 ^a,b^	0.08 ^b^	0.19 ^a,b^	0.30 ^a^	0.071	3.6	0.02
% of time ^1^	0.073	0.006	0.036	0.088			
Object play, √ % of time ^1^	0.62	0.30	0.74	0.57	0.254	1.00	0.40
% of time ^1^	0.38	0.09	0.54	0.32			
Tail position and movement							
Tail low, % of time	58.2	70.3	60.0	61.3	4.15	1.62	0.20
Tail medium/high, √ % of time	3.74	3.15	3.35	3.89	0.397	0.37	0.78
% of time	14.0	9.93	11.2	15.1			
Tail movement, % of time	8.11 ^a,b^	5.30 ^b^	5.45 ^b^	10.10 ^a^	1.659	3.59	0.02
Tail still, % of time	85.9	87.6	87.3	81.4	2.42	2.08	0.12
Location in kennel							
Front, √ % of time	4.23	4.70	3.51	4.90	0.501	2.10	0.12
% of time	17.9	22.1	12.3	24.0			
Back, % of time	39.1	38.1	36.0	35.2	5.78	0.15	0.93
Crate, √ % of time	4.35	3.86	5.45	3.80	0.640	1.49	0.23
% of time	18.9	14.9	29.7	14.5			
Middle, √ % of time	1.51	2.41	1.52	1.97	0.438	1.94	0.14
% of time	2.27	5.81	2.32	3.87			

^1^ Model without day; Means that do not share a superscript letter are significantly different (*p* < 0.05) by Tukey’s test.

**Table 3:** The behaviour of dogs (*n* = 60) exposed to Lavender, Music, DAP or a Control treatment during the post-treatment period.

Behaviour	Lavender	Music	DAP	Control	SED	F-Value	*p*-Value
Activity							
Lie down total, % of time	52.6	58.5	57.4	44.5	4.29	2.79	0.05
Lie down-head down, % of time	42.2	45.3	42.3	35.7	4.35	1.98	0.13
Lie down-head up, √ % of time	2.97	3.19	3.46	2.66	0.411	1.01	0.40
% of time	8.84	10.2	11.9	7.05			
Stand, % of time	32.2	30.2	30.6	38.2	3.47	1.84	0.16
Walk, √ % of time	2.44	2.37	2.35	2.83	0.198	1.00	0.40
% of time	5.94	5.62	5.53	8.00			
Standing exit door, √ % of time	1.08	0.74	0.71	1.61	0.206	2.42	0.08
% of time	1.16	0.55	0.51	2.60			
Sit, √ % of time	1.63	0.83	1.38	1.18	0.334	1.12	0.35
% of time	2.67	0.68	1.92	1.40			
Vocalisation							
Vocalisation, √ % of time	1.52 ^b^	1.56 ^a,b^	1.40 ^b^	2.49 ^a^	0.312	3.89	0.02
% of time	2.32	2.45	1.96	6.21			
Other behaviours							
Body shake, events per hour^1^	1.25	0.96	1.25	1.52	0.549	0.33	0.80
Body scratch, √ % of time ^1^	0.12	0.11	0.27	0.14	0.088	1.53	0.22
% of time ^1^	0.014	0.011	0.075	0.019			
Sniff ground, √ % of time ^1^	0.32 ^a,b^	0.16 ^b^	0.63 ^a^	0.50 ^a,b^	0.142	4.01	0.01
% of time ^1^	0.10	0.025	0.39	0.25			
Groom, √ % of time ^1^	0.80	0.51	0.80	0.51	0.162	2.08	0.12
% of time ^1^	0.64	0.26	0.63	0.26			
Drink, √ % of time	0.42 ^a,b^	0.34 ^b^	0.50 ^a,b^	0.57 ^a^	0.163	2.86	0.05
% of time	0.18	0.12	0.25	0.32			
Pant, √ % of time ^1^	0.82 ^b^	0.91 ^a,b^	1.31 ^a,b^	2.14 ^a^	0.477	3.13	0.04
% of time ^1^	0.68	0.84	1.71	4.58			
Yawn, √ events per hour ^1^	0.48	0.34	0.61	0.38	0.235	0.54	0.66
Events per hour ^1^	0.23	0.11	0.38	0.14			
Excretion √ % of time ^1^	0.21	0.25	0.27	0.27	0.098	0.16	0.92
% of time ^1^	0.045	0.063	0.074	0.074			
Tail position and movement							
Tail low, % of time	61.4	64.7	59.4	67.1	4.01	0.75	0.53
Tail medium/high, √ % of time	3.79	3.60	3.99	4.07	0.389	0.13	0.94
% of time	14.4	13.0	15.9	16.5			
Tail movement, √ % of time	2.54	2.09	2.32	2.97	0.359	1.65	0.19
% of time	6.44	4.36	5.38	8.81			
Tail still % of time	86.7	86.5	86.5	85.9	3.46	0.03	0.99
Location in kennel							
Front, √ % of time	4.61	4.77	4.05	4.49	0.432	0.84	0.48
% of time	21.3	22.8	16.4	20.1			
Back, √ % of time	4.72	5.02	5.07	4.84	0.463	0.18	0.91
% of time	22.2	25.2	25.8	23.4			
Crate, % of time	38.1	32.0	40.5	35.0	5.70	0.56	0.65
Middle, √ % of time	1.12	1.74	1.41	1.53	0.426	0.99	0.41
% of time	1.25	3.02	1.99	2.34			

^1^ Model without day; Means that do not share a superscript letter are significantly different (*p* < 0.05) by Tukey’s test.

**Table 4:** The behaviour of dogs (*n* = 60) exposed to Lavender, Music, DAP or a Control treatment during the Night period.

Behaviour	Lavender	Music	DAP	Control	SED	F-Value	*p*-Value
Activity							
Lie down total, % of time	82.9	83.7	82.0	80.0	2.74	1.06	0.38
Lie down-head down, % of time	78.3	79.5	73.7	76.4	3.15	1.76	0.17
Lie down-head up, √ % of time	1.92	1.70	2.41	1.63	0.336	2.23	0.10
% of time	3.70	2.88	5.81	2.65			
Stand, √ % of time	2.81	2.99	3.10	3.24	0.344	1.07	0.37
% of time	7.89	8.91	9.63	10.5			
Walk, √ % of time	1.29	1.24	1.27	1.41	0.163	0.39	0.76
% of time	1.67	1.54	1.61	1.99			
Standing exit door, √ % of time	0.65	0.45	0.43	0.79	0.166	1.41	0.25
% of time	0.42	0.20	0.18	0.62			
Sit, √ % of time	1.63	0.90	1.53	1.31	0.296	1.91	0.14
% of time	2.65	0.81	2.33	1.71			
Vocalisation							
Vocalisation, √ % of time	1.03	1.24	1.50	1.90	0.281	2.7	0.06
% of time	1.06	1.53	2.24	3.61			
Other behaviours							
Body shake, √ events per hour ^1^	1.40	1.28	1.73	1.31	0.230	1.55	0.22
Events per hour ^1^	1.97	1.65	2.98	1.71			
Sniff ground, √ % of time ^1^	0.24	0.17	0.27	0.27	0.097	0.44	0.73
% of time ^1^	0.057	0.030	0.075	0.073			
Groom, √ % of time	1.07 ^a^	0.61 ^b^	1.01 ^a,b^	0.66 ^a,b^	0.297	4.01	0.01
% of time	1.14	0.37	1.03	0.43			
Excretion, √ % of time ^1^	0.33	0.29	0.32	0.27	0.103	0.12	0.95
% of time ^1^	0.11	0.09	0.10	0.07			
Object play, √ % of time ^1^	0.26	0.05	0.40	0.21	0.138	2.14	0.11
% of time ^1^	0.07	0.003	0.16	0.04			
Tail position and movement							
Tail low, % of time	83.4	85.6	80.0	83.9	2.84	1.91	0.14
Tail medium/high, √ % of time	2.06	2.24	2.34	2.47	0.371	0.38	0.77
% of time	4.22	5.02	5.49	6.10			
Tail movement, √ % of time	1.30	1.38	1.58	1.64	0.293	0.69	0.56
% of time	1.68	1.90	2.48	2.67			
Tail still, % of time	94.3	93.7	91.8	93.6	1.68	0.93	0.44
Location in kennel							
Front, √ % of time ^1^	3.06	3.15	2.64	2.36	0.507	1.02	0.39
% of time ^1^	9.36	9.91	6.96	5.59			
Back, √ % of time	3.28	3.07	3.69	3.40	0.487	0.42	0.74
% of time	10.7	9.41	13.6	11.5			
Crate, % of time ^1^	63.0	70.0	67.3	74.1	8.16	0.66	0.58

^1^ Model without day; Means that do not share a superscript letter are significantly different (*p* < 0.05) by Tukey’s test.

## Appendix A

**Table A1:** Track list.

Artist	Name	Energy (0.0 to 1.0)	Tempo (beats/min)	Valence (0 to 1.0)
Joseph Nagy	Album for the Young, Op. 68: No. 34, Theme in C Major	0.00654	66.018	0.221
Joseph Nagy	Album for the Young, Op. 68: No. 38, Wintertime I in C Minor	0.00918	67.839	0.336
Joseph Nagy	Scenes from Childhood, Op. 15: No. 1, Of Foreign Lands and Peoples in G Major	0.00361	65.28	0.179
Joseph Nagy	Scenes from Childhood, Op. 15: No. 7, Dreaming in F Major	0.00574	69.104	0.36
Joseph Nagy	Scenes from Childhood, Op. 15: No. 12, Child Falling Asleep in E Minor	0.00635	64.76	0.197
Joseph Nagy	Scenes from Childhood, Op. 15: No. 16, The Poet Speaks in G Major	0.00144	66.287	0.352
Felix Mendelssohn	Lieder ohne Worte, Op. 62: No. 1 in G Major	0.0288	62.641	0.0805
Felix Mendelssohn	Lieder ohne Worte, Op. 30: No. 6	0.0151	66.15	0.233
Ludwig van Beethoven	Piano Sonata No. 14 in C-Sharp Minor, Op. 27 No. 2 “Moonlight Sonata”: I. Adagio sostenuto	0.00198	65.771	0.272
Claude Debussy	Deux arabesques, L. 66: No. 1 in E Major, Andantino con moto	0.00374	61.764	0.0872
Ludwig van Beethoven	Bagatelle No. 25 in A Minor, “Fur Elise”	0.00759	67.039	0.315
Claude Debussy	Preludes, Premier livre, L. 117: No. 8, La fille aux cheveux de lin.	0.00166	61.121	0.0594
Claude Debussy	Reverie, L. 68	0.0103	66.779	0.0429
Edvard Grieg	Lyric Pieces, Op. 68: No. 5, At the Cradle	0.0292	67.436	0.138
Edvard Grieg	Lyric Pieces, Op. 71: No. 5, Halling	0.0222	68.266	0.0515
Franz Liszt	Liebestraume, S. 541: No. 3 in A-Flat Major	0.0245	59.104	0.0354
Francis Poulenc	Suite francaise d’apres Claude Gervaise, FP 80: No. 2, Pavane	0.0095	68.753	0.133
Frederic Chopin	Etudes, Op. 10: No. 3 in E Major	0.0469	68.178	0.0395
S. Sound Philharmonic Orchestra	Nocturne, in B-Flat Minor, Op. 9 No. 1	0.0383	66.839	0.0392
S. Sound Philharmonic Orchestra	Nocturne, in D-Flat Major, Op. 27 No. 2	0.0993	63.969	0.12
S. Sound Philharmonic Orchestra	Ballade, in G Minor, Op. 23 No. 1	0.0872	65.974	0.0388
S. Sound Philharmonic Orchestra	Mazurka, in A Minor, Op. 68 No. 2	0.0062	66.343	0.0381
Frederic Chopin	Piano Sonata No. 2 in B-Flat Minor, Op. 35: I. Grave. Doppio movimento	0.0759	61.841	0.0511
S. Sound Philharmonic Orchestra	Waltz No. 3, in A Minor, Op. 34 No. 2	0.0483	66.775	0.0762
S. Sound Philharmonic Orchestra	Waltz, in C-Sharp Minor, Op. 64 No. 2	0.102	68.028	0.183
S. Sound Philharmonic Orchestra	Waltz, in A-Flat Major, Op. 69 No. 1	0.0381	58.053	0.101
Frederic Chopin	Barcarolle in F-Sharp Major, Op. 60	0.0409	57.066	0.0697
Claude Debussy	12 Etudes, L. 136: No. 11	0.0307	65.458	0.0906
Claude Debussy	Estampes: No. 2. La soiree dans Grenade	0.0524	64.25	0.0385
Study Zone	Music for Studying	0.0487	61.996	0.286
Study Zone	Exam Music	0.0398	62.95	0.24
Study Zone	Studying Music	0.0562	65.294	0.246
Study Zone	Relaxation	0.0951	69.955	0.162
Study Zone	Concentration	0.0373	61.146	0.137
Study Zone	Relaxing Song	0.054	68.886	0.12
Study Zone	Musica Relajante	0.0514	61.952	0.0337
Study Zone	Relax	0.104	67.924	0.149
Study Zone	Piano Relajante	0.0358	64.547	0.139
Study Zone	Brain Power	0.0306	59.82	0.113
Michael Nyman	The Scent Of Love	0.0623	64.32	0.116
Michael Nyman	Deep Into The Forest	0.15	67.692	0.113
Calm Children Collection	Lavender Hills	0.141	61.522	0.154
Calm Children Collection	A Single Rose	0.045	59.48	0.0338
Calm Children Collection	Quiet Moves	0.0372	58.487	0.105
Calm Children Collection	Solitary Hill	0.11	69.232	0.0714
Calm Children Collection	Wishing Well	0.0896	56.726	0.153
Calm Children Collection	Light In the Window	0.0885	62.246	0.0435
Calm Children Collection	Fly Away	0.142	65.707	0.0546
Calm Children Collection	Morning Light	0.129	49.689	0.091
James Williams	The Old Windmill	0.0484	68.562	0.0688
Filip Lundqvist	Silent River	0.0259	64.733	0.0895
Frederic Chopin	Nocturne en mi bemol majeur opus 9 nÂ°2: Ballade en Sol Mineur No.1	0.0451	61.494	0.0651
